# Quantification of triglyceride levels in fresh human blood by terahertz time-domain spectroscopy

**DOI:** 10.1038/s41598-021-92656-4

**Published:** 2021-06-24

**Authors:** Dan Wang, Yu Zhang, Juan Han, Xiao Li, Xiaofeng Chen, Tianzhu Qiu, Hua Chen

**Affiliations:** 1 School of Physics, Southeast University, Nanjing, 211189 Jiangsu China; 2 The First Affiliated Hospital, Nanjing Medical University, Nanjing, 210029 Jiangsu China; 3 Nanjing PuKou Central Hospital, Nanjing Medical University, Nanjing, 211800 Jiangsu China

**Keywords:** Biotechnology, Optics and photonics

## Abstract

We conducted a pilot clinical study to investigate ex vivo fresh human blood from 93 patients with coronary heart disease (CHD). The results indicated that terahertz (THz) time-domain spectroscopy (TDS) can be used to quantify triglyceride (TG) levels in human blood. Based on the TG concentrations and corresponding THz absorption coefficients, the Pearson correlation analysis demonstrated that the THz absorption coefficients have a significant negative linear correlation with TG concentration. Comparisons between the THz measurements at 0.2 THz and an automatic biochemical analyzer were performed using an additional 20 blood samples, and the results confirmed that the relative error was less than 15%. Our ex vivo human blood study indicates that the THz technique can be used to assess blood TG levels in clinical diagnostic practice.

## Introduction

TGs are fat molecules formed by long-chain fatty acids and glycerol and are the most abundant lipids in the human body. In patients, high levels of TG are frequently associated with lipid disorders that increase the risk of CHD^1–3^. CHD is a heart disease caused by coronary artery stenosis or obstruction resulting in myocardial ischemia, hypoxia, or necrosis^4^. In recent years, the incidence of CHD has increased. CHD has a high mortality and disability rate, which seriously threatens the life safety of patients and reduces their quality of life^4^. In 2010, the China Chronic Disease Surveillance Study reported that the serum TG levels of people > 18 years of age in 31 provinces were significantly higher than those in 2002 and that the prevalence of TG concentrations ≥ 2.26 mmol/L was 13.8% in males and 8.6% in females^5,6^. In China, based on fasting plasma TG levels, the TG concentration was defined as follows: normal level: < 1.7 mmol/L; high level: 1.7 ~ 2.3 mmol/L; and critical high level: > 2.3 mmol/L^7,8^. In a clinical setting, 2 mL of fresh hemolysis-free serum was mainly obtained by routine venous blood collection, and the TG concentration is determined by an automatic biochemical analyzer. The instrument uses an enzymatic chemical method^9,10^. Initially, TGs are hydrolyzed by lipase to glycerol and fatty acids. Then, glycerol is phosphorylated with glycerase and adenosine triphosphate to produce dihydroxyacetone phosphate, and hydrogen peroxide generates a red quinone imine pigment. Quinone imine absorbance is proportional to the content of TG in the sample. The concentrations of TG were then measured by comparison with the reference samples. Recently, THz-TDS has been suggested as a feasible and sensitive tool to estimate the collective bending vibrations of the hydrogen bonds of water molecules in aqueous solutions^11–13^. Blood has approximately 80% water and contains various types of polarized solutes. Recent studies indicated that the THz absorption of the blood is highly sensitive to the concentration of various solutes since a polarized solute changes the collective bending vibration of the hydrogen bonds^14–17^. In the whole blood system, TG is a dominant factor for THz absorption and is significantly negatively correlated with the THz absorption coefficient^16,17^. Comparisons with existing enzymatic colorimetric assay methods suggest that THz waves can be a potential candidate to monitor the concentrations of TGs in human blood.

In this work, we conducted a pilot clinical THz study to quantify the concentrations of TGs in ex vivo fresh human blood samples from CHD patients. Ten normal and 93 patients’ blood samples were directly assayed by THz-TDS, and the calculated absorption spectra from 0.2 THz to 0.9 THz indicated substantial differences in the THz absorption properties of the human blood samples of different patients, suggesting that the THz absorption coefficients of the blood vary depending on the TG levels. The results of the Pearson correlation analysis indicated that the THz absorption coefficients have a significant negative linear correlation with TG levels. The use of THz-TDS to quantify TG levels without any other methods was confirmed by a comparative experiment between the THz assay and an automatic biochemical analyzer in the blood of an additional group of 20 CHD patients. Our investigation demonstrates that THz waves can potentially be used in clinical diagnostics to assay TG levels in CHD patients.

## Methods

### Clinical protocol

This study was conducted according to the Declaration of Helsinki Principles and approved by the Ethics Committee (IRB) of Southeast University and the First Affiliated Hospital, Nanjing Medical University (No. 3207027381). Informed consent was obtained from each subject prior to study entry. Blood samples from 123 persons in this study were provided by the First Affiliated Hospital, Nanjing Medical University. In the blood spectra acquisition study, we collected 103 persons’ blood samples, including 10 normal persons (mean age: 39 years; range: 33–61 years) and 93 patients with CHD. Among these 93 patients, 38 patients (mean age: 49 years; range: 34–61 years) had high TG concentrations, and 55 patients (mean age: 41 years; range: 44–73 years) had critically high TG concentrations. In the comparative study, we collected an additional 20 patients’ blood samples (mean age: 45 years; range: 36–66 years).

In clinical pathology, CHD does not directly lead to diabetes, but the complications of CHD can lead to diabetes. CHD causes high blood lipids and high blood pressure, which easily leads to abnormal glucose metabolism and bad blood circulation, which can cause diabetes. In addition, coronary artery disease caused by coronary artery stenosis will aggravate abnormal glucose metabolism, thus aggravating the symptoms of diabetes. To exclude the influence of abnormal blood glucose, each subject had a fasting blood glucose test before blood collection, and patients with CHD complicated with diabetes were not included in this study.

### Sample preparation

**Figure 1:**
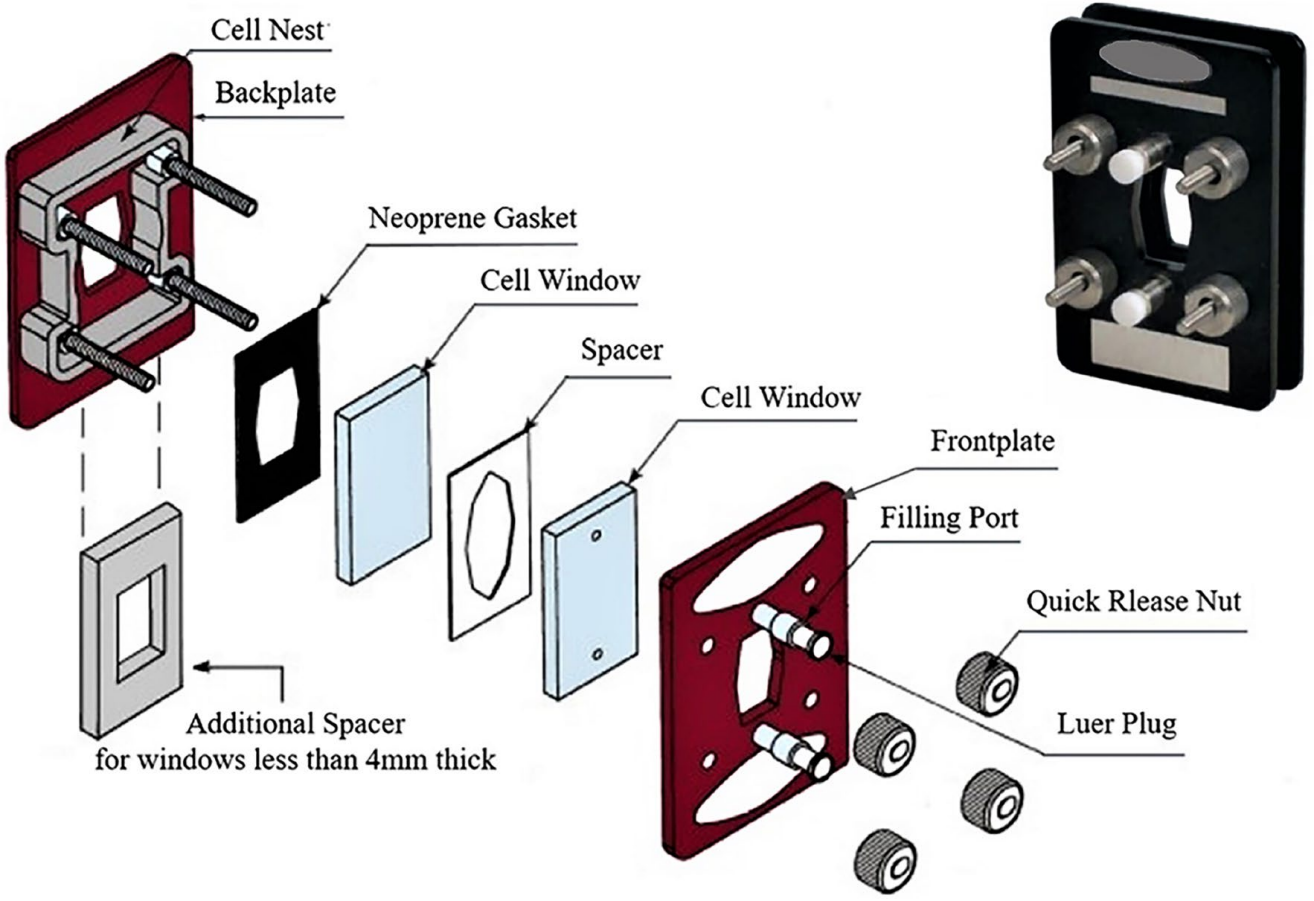
Specac’s Omni cell system^19^.

Immediately after withdrawal, the blood samples were supplemented with heparin as an anticoagulant in a vacutainer (15 US Pharmacopeia units of heparin per milliliter of blood). Previous THz studies have shown that heparin has no effect on THz measurements^18^. Then, the samples were delivered for THz measurement within half an hour. During delivery, the samples were maintained at ≈4 °C. Before the experiment, the temperature of the samples was increased to room temperature by soaking the vacutainers in 25 °C warm water for 5 min. The blood samples were measured by a Specac Omni Cell System (Specac Limited, UK)^19^, as illustrated in Fig. 1. Blood samples were injected into the liquid sample system with two polythene (PE) cell windows and a 200-μm-thick Teflon chamber from a filling port. The chamber was subsequently locked by two plates and four quickly released nuts. After one blood sample was measured, we cleaned the sample system with 75% medical alcohol, dried in a dryer, and then prepared for the next sample measurement. During THz measurement, the room temperature was kept at 23 °C with a stable humidity of 50%.

### Experimental setup

In this experiment, a standard THz-TDS apparatus for transmission measurements was used. The setup and corresponding experimental data analysis method have been discussed in detail in previous reports^16,17^. In brief, a THz-ray is generated by the (1 0 0)-oriented InAs wafer, which is excited by optical pulses from a mode-locked Ti–sapphire laser (λ = 800 nm, τ_FWHM_ = 100 fs). The repetition rate of the laser is 82 MHz. The THz radiation from the InAs collimates and is focused onto the sample layer by off-axis parabolic mirrors. The beam size falling on the Omni cell is approximately 3 mm. Using another parabolic mirror, the THz pulses transmitted through the sample are again collimated and focused onto a detector, which is an electric-optic (EO) ZnTe crystal. A probe beam derived from the same optical source by a half mirror is used to gate the detector. We generated and detected the THz signal in an atmosphere of nitrogen to remove the influence of water vapor on the spectra. The measured THz waveforms showed a main pulse that came from direct transmission through the PE/blood/PE sample system, but there was also a smaller pulse following the main pulse caused by the internal Fabry-Pérot reflection in the PE windows. In our experiments, the smaller reflected pulses are well separated from the main pulses; these echoes are truncated from the measured THz waveforms by restricting the waveforms within a finite time window.

For each measurement, all acquired sample and reference waveforms were converted into their Fourier transforms *E*_sam_(*ω*) and *E*_ref_(*ω*), respectively. A reference waveform was recorded using an empty chamber, described as:

1 $$E_{{{\text{~ref}}}} (\omega ) = E_{{{\text{THz}}}} (\omega ) \cdot T(\omega )_{{{\text{(PE - air)}}}} e^{{(i\omega \cdot d/c)}} .T(\omega )_{{{\text{(air - PE)}}}}$$

where *E*_THz_(*ω*) is the incident electromagnetic wave’s electric field, *d* is the sample thickness, *ω* is the frequency, and *c* is the speed of light in a vacuum. Therefore, the sample waveform *E*_sam_(*ω*) is:

2 $$E_{{{\text{sam}}}} (\omega ) = E_{{{\text{THz}}}} (\omega ) \cdot T(\omega )_{{{\text{(PE - blood)}}}} \cdot e^{{(i\omega d \cdot n(\omega )/c)}} \cdot e^{{( - \omega d \cdot k(\omega )/c)}} \cdot T(\omega )_{{{\text{(blood - PE)}}}} ,$$

where *n*(*ω*) and *k*(*ω*) are the real and imaginary parts of the complex refractive index, respectively. T(ω)_a-b_ is the transmission coefficient from medium a to medium *b*, the absorption of air is negligible, and the refractive index of air is 1. Therefore, the refractive index and extinction coefficient of the sample can be approximately displayed as follows^20^:

3 $$n_{{{\text{sam}}}} (\omega ) \approx (\Delta \phi ~_{{{\text{sam - ref}}}} ) \times c/\omega d + 1,$$

4 $$k_{{{\text{sam}}}} (\omega ) \approx [\ln (|E_{{{\text{sam}}}} (\omega )/E_{{{\text{ref}}}} (\omega )|^{2} )/d] \times (c/2\omega ),$$

where $\Delta \phi _{{{\text{sam - ref}}}}$ is the phase difference. Then, we obtained the absorption coefficient *α* in the form of 2*·ω·κ*(*ω*)*/c*. To improve the precision and accuracy, we calculated the absorption coefficient *α* and the refractive index *n* by using Lytera’s TeraLyzer software.

## Results

### THz absorption spectra of the blood

**Figure 2:**
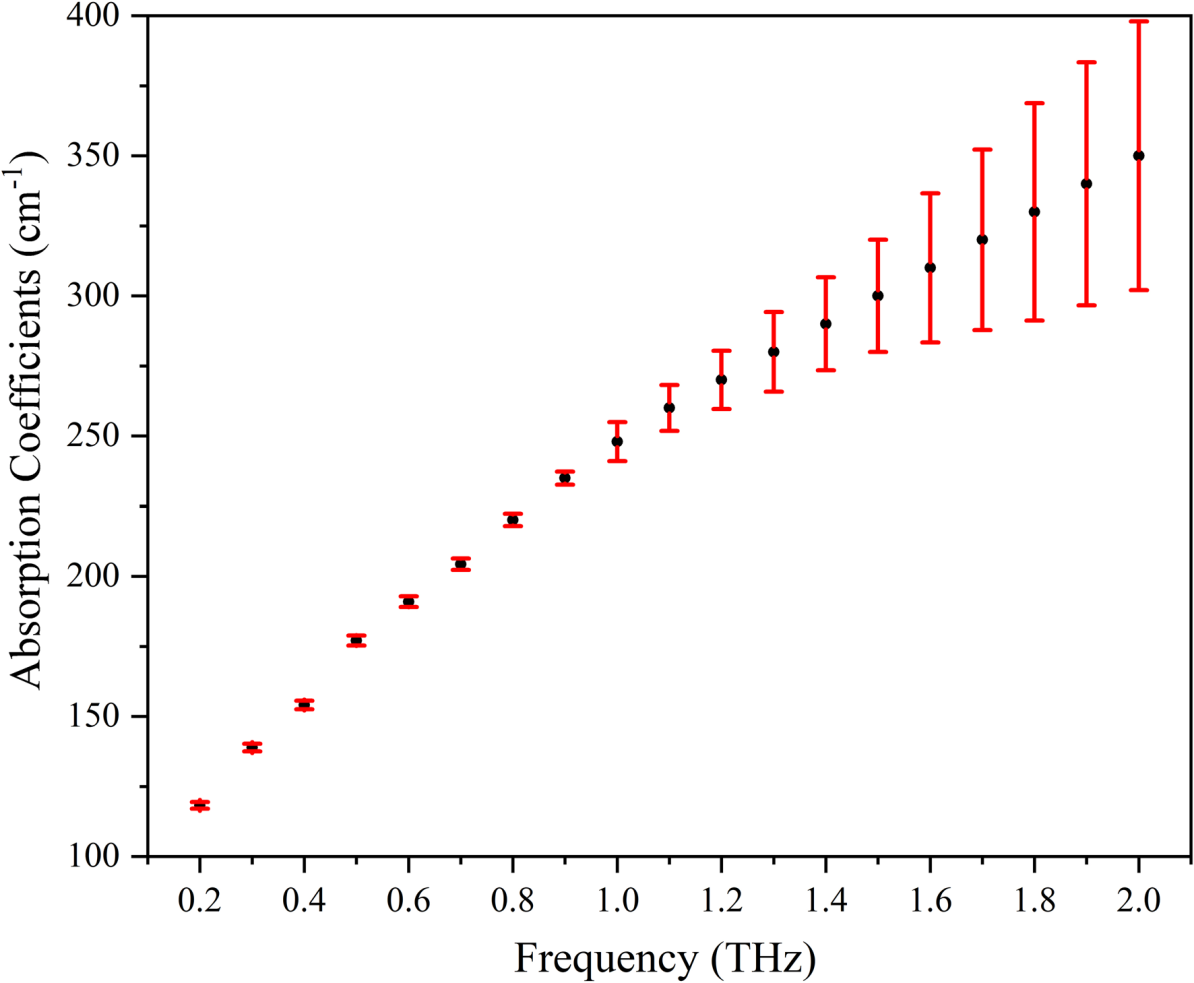
Five measured absorption coefficients of pure water. The red error bars represent the standard deviation of the mean, and the test number is 5.

Before measuring the blood, we first measured pure water (tap water) with this liquid sample system for a repeatability test, and the test number was five. The repeatability test included the assembling the sample system, fixing the system, injecting water, disassembling, cleaning and drying the system; it also included removing and repositioning the sample system in a THz beam. Figure 2 shows the five measured absorption coefficient spectra of water. The results indicated that our measurement has a high repeatability in the frequency range between 0.2 and 0.9 THz, the ratio of the standard deviation to the average value was as low as 1%, and the high water absorption made the data unreliable at higher THz frequencies.

**Figure 3:**
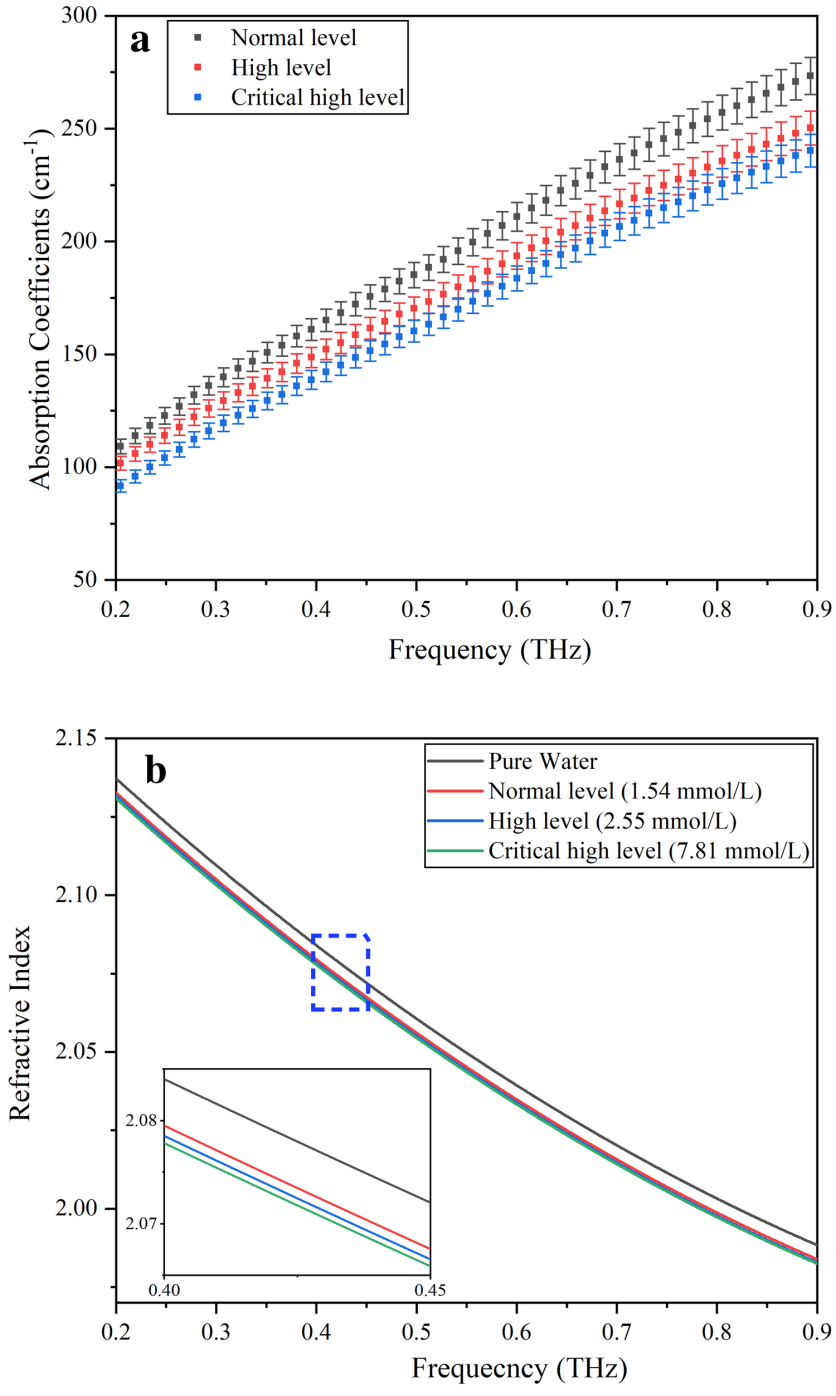
(**a**) THz absorption coefficient spectra of three groups of blood samples (normal level: 10; high level: 38; critical high level: 55). The solid squares represent the mean, and the error bars represent the deviation of the mean. (**b**) Refractive index of water and 3 whole blood samples.

For the blood sample study, we repeated every blood sample measurement five times by removing and repositioning the sample system in the beam, and the ratio of the standard deviation to the average value was < 1% from 0.2 THz to 0.9 THz. Figure 3a shows the absorption coefficient spectra of 103 blood samples (10 normal persons’ blood, 38 high-level CHD patients’ blood and 55 critical high-level CHD patients’ blood). For clarity, the three groups of data are presented in the form of the mean and mean deviation. As shown in Fig. 3a, it is clear that the absorption coefficients of all blood samples increase when the THz frequency increases, which means that the main effect is the absorption of water. Meanwhile, substantial differences in the THz absorption coefficients of human blood were detected between persons in different TG concentration situations, and blood with higher TG levels caused lower THz absorption. In Fig. 3b, we also summarized the refractive indices of pure water and whole blood. Three different TG concentrations were used for the blood samples. It is clear that the whole blood samples have refractive indices very similar to pure water. Compared to pure water, the refractive index of whole blood is smaller. We think the lower refractive index is because water molecules may weakly bond to polar functional groups on the surface of TG and the weak bonding of bound water modifies the vibrations of the water molecules.

### Correlation analysis

To investigate the correlation of the TG levels and THz absorption, we used Pearson correlation analysis to estimate the correlations between the THz absorption coefficient and TG levels. According to the Pearson correlation analysis method, the correlation coefficient *R* is defined as^21^:

5 $$R = \frac{{\sum\nolimits_{{i = 1}}^{n} {\left( {x_{i} - \bar{x}} \right)\left( {y_{i} - \bar{y}} \right)} }}{{({\text{n}} - 1)S_{x} S_{y} }} = \frac{{\sum\nolimits_{{i = 1}}^{n} {\left( {x_{i} - \bar{x}} \right)\left( {y_{i} - \bar{y}} \right)} }}{{\sqrt {\sum\nolimits_{{i = 1}}^{n} {\left( {x_{i} - \bar{x}} \right)^{2} \sum\nolimits_{{i = 1}}^{n} {\left( {y_{i} - \bar{y}} \right)^{2} } } } }},\quad i = 1,2,3 \ldots n$$

In the process of analysis, we set *x* as the absorption coefficient and *y* as the concentration of TG in the human blood samples obtained from the results of the enzymatic colorimetric assay. If *R* > 0, the correlation is positive, and if R < 0, the correlation is negative. The absolute value of *R* (|*R*|) is a measure of the strength of the linear relationship between the *x* and *y* values of a data pair. If |*R*| is equal to 1, there is a perfect linear relationship, and a straight line can pass through all data points. If *R* > 0.5, there is a strong correlation. Finally, according to Eq. (5), we calculated the *R* values at various THz frequencies by using the absorption coefficients shown in Fig. 3a and TG concentrations from the enzymatic colorimetric assay. To verify the accuracy of the results, Student’s t-test was used to compare the data, and the *p*-value was obtained using Statistical Product and Service Solutions (SPSS) software^22^. If the *p*-value is less than 0.05, the THz absorption coefficient is considered to be related to the TG concentration^23^.

**Figure 4:**
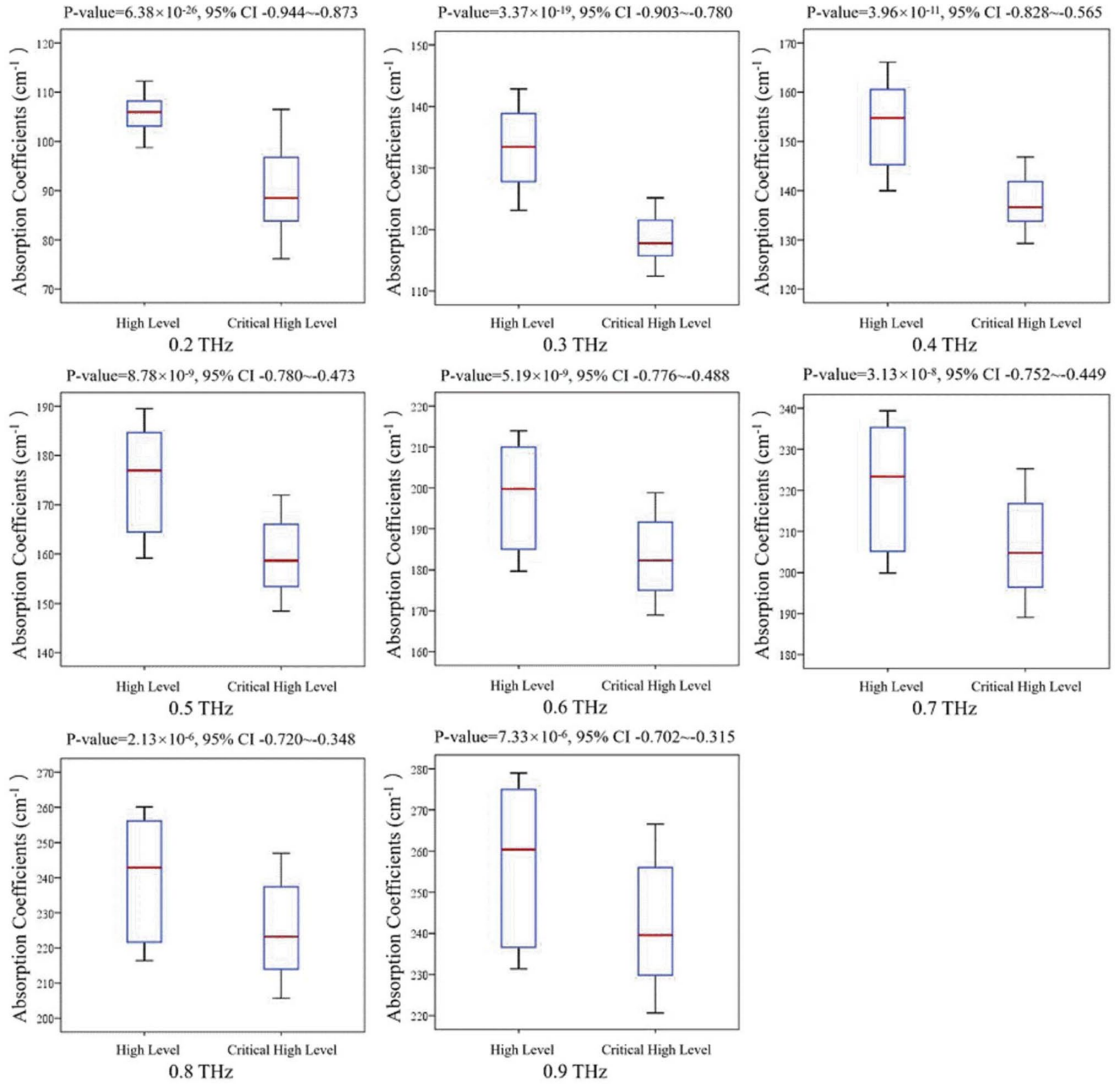
Two-tailed unpaired t-test between the THz absorption coefficients of the high-level group (n = 38) and critical high-level group (n = 55). The central red line indicates the median, the bottom and top edges of the box indicate the upper and lower quartiles, and the whiskers extend to the most extreme data points not considered outliers. *P*-values and confidence intervals (95% CI) for the difference in two groups are also denoted.

The values of *R* and the corresponding *p*-values from 0.2 THz to 0.9 THz are shown in Table 1. The data in Table 1 indicate that all |*R*| values are greater than 0.5 and that all *R* values are negative. The results were also validated by a two-tailed unpaired T-test. We performed a two-tailed unpaired T-test between the THz absorption coefficients of the high-level group (TG concentration < 2.3 mmol/L, n = 38) and critical high-level group (TG concentration > 2.3 mmol/L, n = 55), as shown in Fig. 4. The unpaired T-test indicates that the THz absorption coefficient is highly coherent with the concentration of TG and that the absorption coefficient decreases concomitant with an increase in the TG levels. In the whole human blood system, cholesterol and fatty acids are the most dominant lipids. TGs are essential blood lipid esters derived from glycerol and three fatty acids. Therefore, we conclude that the negative correlation between the TG concentrations and THz absorption is because lipids absorb considerably lower amounts of THz power than those absorbed by water.

**Table 1:** Results of statistical analysis results summarizing the *p*-value and Pearson correlation coefficient between the THz absorption and the TG concentrations.

Frequency (THz)	*R*	*P*-value
0.2	− 0.914	6.38 × 10^−26^
0.3	− 0.853	3.37 × 10^−19^
0.4	− 0.713	3.96 × 10^−11^
0.5	− 0.645	8.78 × 10^−9^
0.6	− 0.652	5.19 × 10^−9^
0.7	− 0.626	3.13 × 10^−8^
0.8	− 0.553	2.13 × 10^−6^
0.9	− 0.528	7.33 × 10^−6^

### Quantitative analysis

According to a previous whole human blood study by Sun^16,17^, other components could also modify the THz absorption coefficients. First, we performed a statistical analysis based on the THz coefficient and various factors in whole blood, including glucose, Na^+^, Ca^2+^, K^+^, red blood cells (RBCs), hemoglobin (HB), and white blood cells (WBCs). Corresponding concentration information was obtained from 75 blood test reports (blood from 8 normal patients, blood from 36 high-level CHD patients, and blood from 31 critical high-level CHD patients). In Table 2, we show the values of *R* and the corresponding *p*-values, and the results indicate that the RBC has a significant negative correlation with the THz coefficient in the low sub-THz region at 0.2 THz, while the concentrations of other factors are not correlated with the absorption coefficient.

**Table 2:** Results of statistical analysis results summarizing the Pearson correlation coefficient and the *p*-value between the THz absorption and examination items.

Items	*R* and *p*-value		
	0.2 THz	0.5 THz	0.8 THz
Glucose (3.89–6.11) mmol/L	R = − 0.135, *p* = 0.243	R = − 0.232, *p* = 0.256	R = − 0.265, *p* = 0.248
RBC (3.5–5.5) 10^12^/L	R = − 0.453, *p* = 0.0052	R = − 0.231, *p* = 0.085	R = − 0.034, *p* = 0.278
Na^+^ (135–145) mmol/L	R = 0.274, *p* = 0.135	R = 0.127, *p* = 0.153	R = 0.137, *p* = 0.259
Ca^2+^ (2.1–2.6) mmol/L	R = 0.114, *p* = 0.569	R = − 0.045, *p* = 0.524	R = − 0.215, *p* = 0.431
K^+^ (3.5–5.3) mmol/L	R = 0.298, *p* = 0.432	R = 0.178, *p* = 0.286	R = − 0.169, *p* = 0.179
HB (110–160) g/L	R = − 0.135, *p* = 0.243	R = − 0.135, *p* = 0.243	R = − 0.135, *p* = 0.243
WBC (4.0–10.0) 10^9^/L	R = − 0.390, *p* = 0.189	R = − 0.255, *p* = 0.263	R = − 0.103, *p* = 0.773

The range of each item is listed in parenthesis.

**Figure 5:**
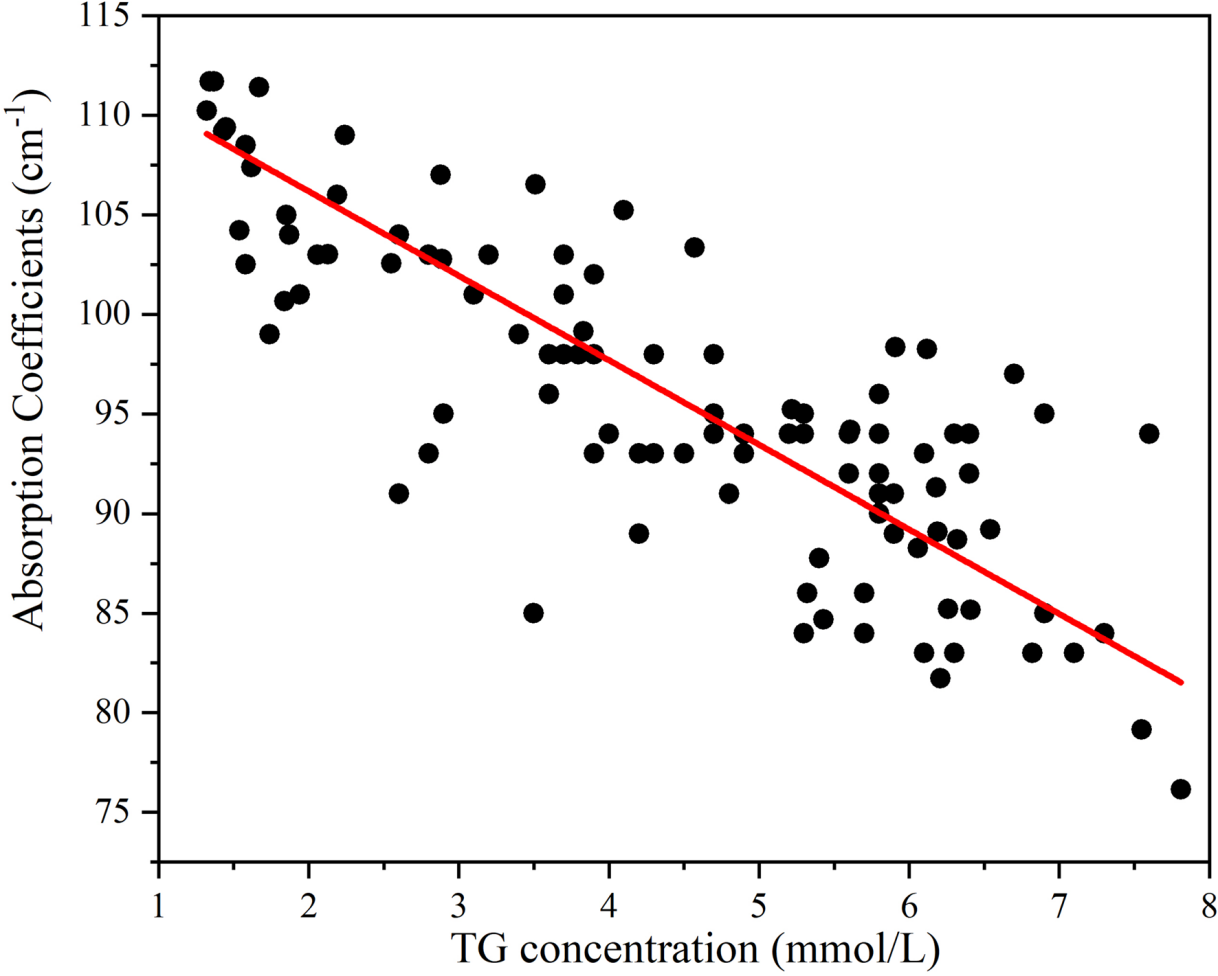
The correlation between the THz wave absorption coefficients and TG concentrations in human blood.

Moreover, TG has the highest |*R*| at 0.2 THz. The water content is the most dominant factor in the absorption of THz power by human blood, and the THz absorption of water decreases at lower frequencies. Therefore, we investigated the relationship between the THz absorption coefficients at 0.2 THz and TG concentrations by multiple linear regression analysis. The data on 103 blood samples shown in Fig. 5 indicate that the THz absorption coefficients and the concentrations of TG in the blood have a linear relationship: *α* = 116.72 − 4.13*C*_TG_ − 3.21*C*_RBC_, where *C* corresponds to the concentrations of certain human blood components. This linear correlation indicates that quantitative TG level analysis using THz-TDS is feasible.

Based on this linear relationship, we attempted to test direct quantification of the TG levels by THz-TDS. An additional 20 blood samples were selected and divided into two parts. One part was used in the THz-TDS measurements, and another part was used for automatic biochemical analyzer measurements. We performed these measurements separately, meaning that before the THz-TDS measurements, we did not know the blood TG contents in these 20 samples. After we measured 20 blood samples by THz-TDS and calculated the corresponding absorption coefficients at 0.2 THz, we calculated the TG contents according to the linear relationship of *α* = 116.72 − 4.13*C*_TG_ − 3.21*C*_RBC_. Then, we measured 20 blood samples by an automatic biochemical analyzer. Finally, we compared two sets of results in Table 3. It is clear that the results from these two measurements are similar. A comparison of the results of these two measurements indicates that the relative error is less than 15%. Therefore, the results demonstrate that THz-TDS can be used to quantify TG levels in human blood as a stand-alone method.

**Table 3:** TG contents were measured by THz-TDS and an automatic biochemical analyzer.

Methods	Relative error (%)	
	THz-TDS (mmol/L)	Automatic biochemical analyser (mmol/L)
4.63	4.16	11.3
5.41	4.84	11.8
5.72	6.17	7.29
5.58	6.04	7.62
5.57	4.89	13.9
5.58	5.04	10.7
5.68	5.14	10.5
6.31	5.49	14.9
6.10	5.35	14.0
6.06	5.47	10.8
6.26	5.63	11.2
6.31	5.62	12.3
6.51	5.89	10.5
6.79	6.03	12.6
7.12	6.28	13.4
3.84	4.46	13.9
3.42	3.21	6.54
2.92	3.32	12.0
1.87	2.03	7.88
1.85	1.71	8.19

### Discussion

At present, chemometric analysis^9,10^ is used as the clinical analysis method for CHD; this method requires venous blood sampling for detection, which makes it difficult to avoid the risk of pain and infection. Moreover, the feedback based on the results of the analysis is slow. Therefore, this method cannot be used for noninvasive continuous real-time monitoring. Fourier transform infrared spectroscopy (FTIR)^24–26^ and Raman spectroscopy^24,27^ are widely used in pilot clinical studies to assay TG content in human blood based on the parameters of vibration and the energy levels of the molecules. However, it is difficult to detect high-quality low-frequency vibration spectra and the corresponding molecular information. In biological macromolecules^28,29^, the absorption frequencies correspond to the low frequency stretching and bending vibration between the molecules and within molecules; the phonon vibration of lattices and the stretching and torsion vibration of the hydrogen bonds are distributed in the THz band. In addition, 80% of the blood is water, and the contents of glucose, cholesterol, and TGs are approximately 0.1%. In aqueous solutions, THz waves have been shown to be a sensitive tool for investigating the bending vibrations of the hydrogen bond groups of water molecules^11–13^. THz absorption of an aqueous solution is highly sensitive to the concentrations of various solutes because polarized solutes can change the collective bending vibration of hydrogen bonds; the strong absorption of THz waves by water molecules is compatible with the nondestructive detection of the water content and distribution. Moreover, the photon energy of the THz wave is only a few parts per million of the X-ray photon energy, which is lower than the bond energy of various chemical bonds^30^. Therefore, THz radiation will not lead to photoionization nor harm the human body, which is suitable for in vivo detection in the human body^31^. Due to its capability to perform quantitative analysis and enable follow-up traditional blood pathological analysis, our study indicated considerable potential of THz-TDS for future rapid, accurate, and continuous blood examinations.

### Conclusions

In conclusion, the THz absorption coefficient spectra of the fresh blood samples from 93 patients with CHD disease indicated that the TG concentration is a dominant factor that influences the whole blood absorption coefficient in the THz region from 0.2 THz to 0.9 THz and that the TG concentration has a significant negative correlation with the THz absorption coefficient. A linear relationship between the TG absorption coefficient and the concentrations of TG was detected. The use of THz-TDS to directly quantify TG levels was also demonstrated as a stand-alone method. Comparison of the results to the data of an automatic biochemical analyzer indicates that the relative error is less than 15%. Our study provides a method for rapid, accurate, and continuous measurement of blood TG levels in patients with CHD, which has great potential in clinical applications.

## Data Availability

The data that support the findings of this study are available on request from the corresponding author.
